# hsa_circ_0026827 Promotes Osteoblast Differentiation of Human Dental Pulp Stem Cells Through the Beclin1 and RUNX1 Signaling Pathways by Sponging miR-188-3p

**DOI:** 10.3389/fcell.2020.00470

**Published:** 2020-06-26

**Authors:** Fang Ji, Lanying Zhu, Jing Pan, Zhecheng Shen, Zhao Yang, Jian Wang, Xuebing Bai, Yueting Lin, Jiang Tao

**Affiliations:** ^1^Department of Orthodontics, Ninth People’s Hospital, College of Stomatology, Shanghai Jiao Tong University School of Medicine, Shanghai, China; ^2^National Clinical Research Center for Oral Diseases, Shanghai Key Laboratory of Stomatology and Shanghai Research Institute of Stomatology, Shanghai, China; ^3^Department of Stomatology, Jining Traditional Chinese Medicine Hospital, Shandong, China; ^4^Department of General Dentistry, College of Stomatology, Ninth People’s Hospital, Shanghai Jiao Tong University School of Medicine, Shanghai, China

**Keywords:** dental pulp stem cells, osteoblast differentiation, hsa_circ_0026827, miR-188-3p, Beclin1, RUNX1

## Abstract

Previous studies have found that circular RNA (circRNA) hsa_circ_0026827 plays a role during osteoblast differentiation, but the mechanism is unclear. The aim of this study was to illuminate the role of hsa_circ_0026827 in human dental pulp stem cells (DPSCs) during osteoblast differentiation. The results show that hsa_circ_0026827 expression significantly increased during osteoblast differentiation, while knockdown of hsa_circ_0026827 suppressed DPSC-derived osteoblast differentiation. microRNA (miRNA) expression profile analysis showed that downregulation of hsa_circ_0026827 promoted miR-188-3p expression. miR-188-3p downregulation restored osteogenic differentiation of DPSCs after hsa_circ_0026827 was silenced. Luciferase reporter assays verified that miR-188-3p was the target of hsa_circ_0026827 and also demonstrated that Beclin1 and RUNX1 were miR-188-3p downstream targets. miR-188-3p overexpression suppressed DPSC osteogenic differentiation by targeting Beclin-1-mediated autophagy and runt-related transcription factor 1 (RUNX1). *In vivo* studies using a heterotopic bone model also found that hsa_circ_0026827 overexpression plays an important role in promoting heterotopic bone formation. In conclusion, our research indicates that hsa_circ_0026827 promotes osteoblast differentiation of DPSCs via Beclin1 and the RUNX1 signaling pathways by sponging miR-188-3p, which suggests novel therapeutics for osteoporosis treatment.

## Introduction

Dental pulp stem cells (DPSCs) belong to a class of multipotent mesenchymal stem cells (MSCs) which can differentiate into distinct specialized cell types such as osteocytes, adipocytes and chondrocytes (Mangano et al., 2010; Spath et al., 2010; Wang et al., 2017). It has been reported that DPSCs are more effective in proliferation and osteogenesis and have lower immunogenicity than MSCs (Ching et al., 2017). Dental pulp stem cells may thus be useful in oral and maxillofacial reconstruction (Rapino et al., 2019). During osteoblast differentiation, DPSCs express osteoblast differentiation-related biomarkers such as runt-related transcription factor 1 (RUNX1), osteocalcin (OCN), alkaline phosphatase (ALP), and osterix (OSX) (Sharpe, 2016; Victor and Reiter, 2017). Our previous studies have found that downregulation of nuclear factor erythroid 2 related factor promotes autophagy-dependent osteoblastic adipose-derived MSC differentiation (Tao et al., 2016). Although the regulatory role of coding genes in osteoblast differentiation has been broadly studied, the functional contributions of non-coding RNAs, specifically those of circular RNAs (circRNAs), remain unknown.

circRNAs have been shown to be indispensable in post-transcriptional transcriptome regulation (Hansen et al., 2013; Liu et al., 2017). circRNAs are constructed by specific loop splicing and are resistant to RNase R digestion. In contrast to linear RNAs, circRNAs are constructed as a covalently closed continuous loops with their 5′ tails and 3′ heads bound together (Qu et al., 2015). The loop structure is formed via a specific class of alternative splicing event called back-splicing, in which an upstream splice acceptor is joined to a downstream splice donor (Daniel et al., 2015). circRNAs harbor microRNA (miRNA) binding sites, which normally function as miRNA sponges (Hansen et al., 2013). For example, a previous study has validated that circRNA CDR1as acts as an miR-7 inhibitor, which triggers GDF5 upregulation and subsequent p38 and Smad1/5/8 MAPK phosphorylation to enhance osteogenic differentiation of periodontal ligament stem cells (Li et al., 2018). circIGSF11 silencing increases miR-199b-5p expression and enhances osteoblast differentiation (Zhang et al., 2019). circRNA expression profile analysis has demonstrated that hsa_circ_0026827 is abnormally expressed during osteoblast differentiation, but its function is unclear (Zhang et al., 2019).

Thus current investigation aimed to explore the role of hsa_circ_0026827 in DPSC osteoblast differentiation. Our data suggest candidate therapeutic strategies for regeneration of bone and periodontal tissue and also reveal new mechanisms underlying osteogenic differentiation.

## Materials and Methods

### Ethics Statement

All treatments involving animals were approved by the Animal Care Committee of the Ninth People’s Hospital, Shanghai Jiao Tong University School of Medicine, Shanghai, China.

### DPSC Isolation and Identification

We isolated cells from dental pulp as described in Iezzi et al. (2019). In brief, we gently removed tissue (Gain from Department of Orthodontics, Ninth People’s Hospital, Shanghai Jiao Tong University School of Medicine. The Ethics Committee of Ninth People’s Hospital, Shanghai Jiao Tong University School of Medicine approval the DPSCs isolation) and immersed it in a digestive solution (4.0 mg/ml dispase and 3.0 mg/ml type I collagenase) for 1 h under at 37°C. We then filtered the digested solution with 70-μm cell strainers to obtain an DPSCs suspension. We plated cells in T25 flasks and cultured them in complete culture medium containing DMEM/F12 with fetal bovine serum (FBS; 10%) and penicillin/streptomycin (1%) at 37°C in 5% CO_2_.

We harvested DPSCs in 5 mM ethylene diamine tetraacetic acid (EDTA) in phosphate-buffered saline (PBS) for surface protein flow cytometric analysis. We incubated cells with PE- or FITC-conjugated antibodies against human CD90, CD34, CD29, CD45, CD44, CD73, and CD105 (Becton Dickinson, San Jose, CA, United States). Matched isotype antibodies were employed as controls. We washed cells once with cold PBS containing 2% fetal calf serum. We acquired 1000 labeled cells and analyzed them by a FACScan flow cytometer running CellQuest (Becton Dickinson) (Dominici et al., 2006; Shen et al., 2019).

### Plasmid Construction and Transfection

We purchased overexpression plasmids pcDNA3.1-RUNX1 and pcDNA3.1-hsa_circ_0026827 from GeneChem Co., Ltd. (Shanghai, China). We also purchased siRNA against hsa_circ_0026827 (sicircRNA), miR-188-3p inhibitor and mimic from GeneChem. We performed cell transfections using Lipofectamine^®^ 3000 (Invitrogen Life Technologies, United States) following the manufacturer’s protocol.

### miRNA Microarray Procedures

We used DPSCs for RNA sequencing. We constructed libraries using the Illumina Gene Expression Sample Preparation Kit and sequenced them with the Illumina HiSeqTM 2000 (next generation sequencing) from Beijing Genomics Institute, Beijing, China. In brief, we isolated total RNA from each sample and treated them with DNase I to degrade any potential DNA contamination, then enriched mRNA with oligo (dT) magnetic beads. We mixed enriched mRNA with fragmentation buffer and fragmented it into short fragments (∼200 bp) from which we synthesized the first strand via a random hexamer primer. We synthesized the second strand after adding reaction buffer, RNaseH, dNTPs, and DNA polymerase I to the first strand synthesis mixture. We purified double-stranded cDNA with magnetic beads and added a 3′-terminal single nucleotide adenine. Lastly, we ligated sequencing adaptor to the fragment to amplify it by PCR. We sequenced enriched fragments by the Illumina HiSeq^TM^ 2000 and generated 50-bp raw reads by the Illumina Genome Analyzer II.

### Real-Time Quantitative Reverse Transcription PCR Detection

We extracted total RNA using Trizol reagent (Invitrogen, Carlsbad, CA, United States). We utilized RNase-free DNase Set (Qiagen) to erase genomic DNA contamination. We reverse transcribed 1 μg of total RNA using transcriptase (Applied Biosystems, Foster City, CA, United States) and random primers for cDNA synthesis. Afterward, we performed real-time quantitative reverse transcription PCR (RT-qPCR) utilizing Power SYBR Green PCR Mastermix (Applied Biosystems) on the Applied Biosystems 7500 Real-time Fast PCR System. We carried out PCR in triplicate for every gene. We calculated relative expression by the 2^–ΔΔCt^ method, with glyceraldehyde-3-phosphate dehydrogenase (GAPDH) or U6 for normalization. Table 1 lists human gene-specific PCR primers.

**TABLE 1:** Primers used in this study.

Gene name	Forward (5′-3′)	Reverse (5′-3′)
RUNX1	ACTACCAGCCACCGAGACCA	ACTGCTTGCAGCCTTAAATGA CTCT
OCN	AGCCACCGAGACACCATGAGA	GGCTGCACCTTTGCTGGACT
ALP	GAACGTGGTCACCTCCATCCT	TCTCGTGGTCACAATGC
OSX	ACTGCCCCACCCCTTAGACA	GAGGTGCACCCCCAAACCAA
miR-188-3p	ATGTACACAAGCACACCTTCT CATT	TCAGAAAGCTCACCCTCC ACCAT
hsa_circ_ 0026827	GCTGAAGAATTAAATC	CGAAGTTCCGTCTACGGC
U6	CTCGCTTCGGCAGCACA	AACGCTTCACGAATTTGCGT
GAPDH	CGACAGTCAGCCGCATCTT	CCAATACGACCAAATCCGTTG

### Dual Luciferase Reporter Assay

We generated reporter plasmids by adding circRNA, Beclin-1 or RUNX1 3′-UTR sequence to the pGL3 vector (Promega, Madison, WI, United States). We co-transfected miR-188-3p mimics and reporter plasmids into 239T cells via Lipofectamine 2000 for the luciferase assay. After culturing for two days, we measured firefly and Renilla luciferase activities through the Dual Luciferase Reporter Assay System (Promega) following standard procedures.

### Alkaline Phosphatase Staining

We employed the NBT/BCIP staining kit (CoWin Biotech, Beijing, China) for ALP staining following the manufacturer’s protocol. We seeded DPSCs in 24-well plates and cultured them in osteogenic medium (OM) for one or two weeks. We then fixed cells with 4% paraformaldehyde (PFA) for 30 min, followed by incubation in staining reagent in the dark for 20 min.

### Mineralization Assay

We seeded DPSCs in 24-well tissue culture plates and cultured them for 1 or 2 weeks in OM so as to measure calcium deposition in the extracellular matrix. We employed a solution of 0.1% Alizarin red S (Sigma-Aldrich, Saint Louis, MO, United States) at pH 4.2 to stain the calcified nodules after fixing DPSCs in 4% PFA.

### *In vivo* Heterotopic Bone Formation Assay

We induced DPSCs under OM for 1 week prior to *in vivo* study. We resuspended cells and incubated them with 7 mm × 5 mm × 2 mm Bio-Oss Collagen scaffolds (Geistlich, GEWO GmbH, Baden-Baden, Germany) for 1 h at 37°C followed by centrifugation at 150 g for 5 min. We then implanted them subcutaneously on the backs of BALB/c homozygous nude (nu/nu) mice (5 mice per group) for 5 weeks old according to Jin et al. (2016). We harvested implants after implanted for eight weeks.

### Masson’s Trichrome, H&E Staining and Immunohistochemical Analysis

We decalcified implanted scaffolds in 10% EDTA, pH 7.4 for 1 month, followed by dehydration and embedding in paraffin. We cut sections of heterotopic bones (5 μm) and stained them with Masson’s trichrome and hematoxylin and eosin (H&E). Also, we evaluated sections by immunohistochemical analysis according to Wei et al. (2014). We blocked specimens with 5% normal goat serum for 30 min and then incubated them with primary antibody against OCN (Santa Cruz Biotechnology, Dallas, TX, United States) at 4°C overnight. We then processed sections through an ABC detection kit (Vector Laboratories, Burlingame, CA, United States) and visualized them under an Olympus microscope (Olympus Co., Tokyo, Japan).

### Statistical Analysis

Data are displayed as means ± SD (standard deviation). We utilized GraphPad Prism, version 5.0 (GraphPad, La Jolla, CA, United States) to analyze group differences. *P* ≤ 0.05 was regarded as statistically significant.

## Results

### Expression of hsa_circ_0026827 Increased With Osteogenic Differentiation of DPSCs

**FIGURE 1:**
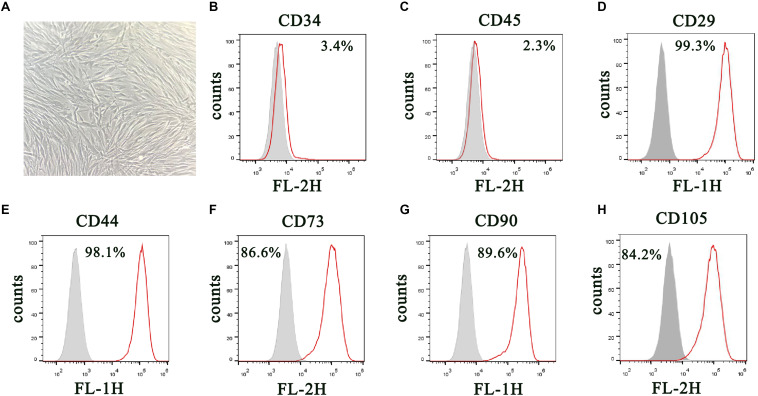
Isolation and characterization of DPSCs. **(A)** Morphology of DPSCs show adoption of fibroblast-like morphology. **(B–H)** Surface protein profiles of DPSCs as analyzed by flow cytometry.

**FIGURE 2:**
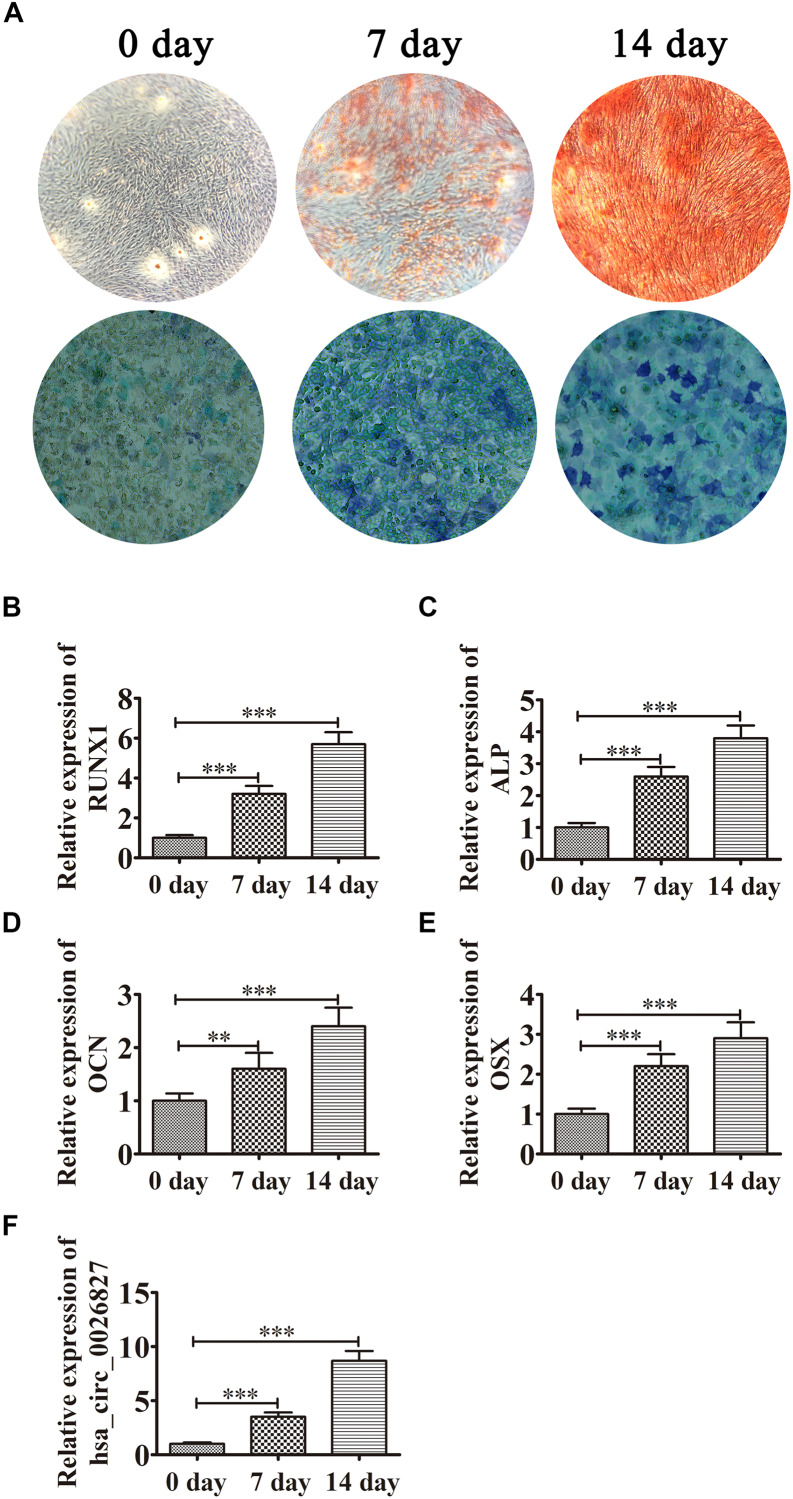
Expression of hsa_circ_0026827 increased with osteogenic differentiation of DPSCs. **(A)** Photographs of ALP (bottom images) and alizarin red staining (top images) show the osteogenic differentiation potential of DPSCs. **(B–E)** RT-qPCR detection shows the expression of RUNX1, OCN, ALP and OSX. Results are presented as means ± SD. ^∗∗^*P* < 0.01, ^∗∗∗^*P* < 0.001 vs control (0 day). **(F)** RT-qPCR detection shows the expression of hsa_circ_0026827. Results are presented as means ± SD. ^∗∗∗^*P* < 0.001 vs control (0 day).

DPSCs were isolated and displayed a typical cobblestone-like morphology (Figure 1A). Immunofluorescence staining showed that isolated DPSCs were negative for CD34 (Figure 1B) and CD45 (Figure 1C) expression but were positive for mesenchymal cell surface markers CD29 (Figure 1D), CD44 (Figure 1E), CD73 (Figure 1F), CD90 (Figure 1G), and CD105 (Figure 1H). The osteogenic potential of DPSCs was analyzed with ALP and Alizarin Red S (ARS) staining. Results showed that odontogenic induction promoted osteogenic differentiation of DPSCs in a time-dependent manner (Figure 2A). RT-qPCR detection showed that mRNA expression of osteogenic markers *RUNX1*, *ALP*, *OSX* and *OCN* was also consistently and significantly increased during osteogenic differentiation (Figures 2B–E).

Previous studies have found that hsa_circ_0026827 expression promotes osteogenic differentiation (Zhang et al., 2019). To determine if hsa_circ_0026827 plays a role in DPSC osteogenic differentiation, we quantified the hsa_circ_0026827 expression level with RT-qPCR and showed that its expression significantly increased during osteogenic differentiation (Figure 2F).

### hsa_circ_0026827 Knockdown Decreased the Osteogenic Differentiation Potential of DPSCs by Regulating miRNA Expression

**FIGURE 3:**
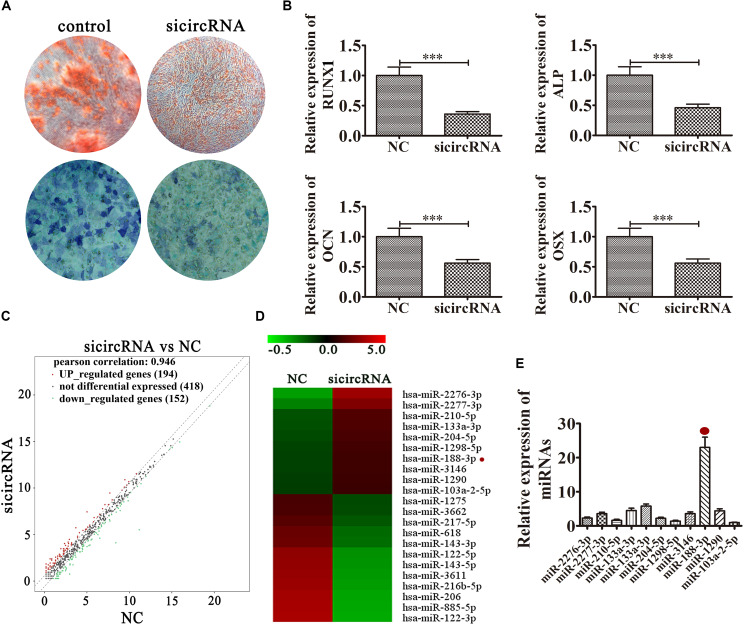
Knockdown of hsa_circ_0026827 decreased the osteogenic differentiation potential of DPSCs by regulating miRNA expression. **(A)** Photographs of ALP (bottom images) and alizarin red staining (top images) show the osteogenic differentiation potential of DPSCs after induction for 14 days. **(B)** RT-qPCR detection shows the expression of RUNX1, OCN, ALP, OSX. Results are presented as means ± SD. ^∗∗∗^*P* < 0.001 vs control. **(C)** Hierarchical cluster analysis of the samples found that a total of 194 miRNAs were upregulated and 152 miRNAs were downregulated after hsa_circ_0026827 knockdown in DPSCs. **(D)** Heat map from microarray data showing the differentially expressed miRNAs. **(E)** RT-qPCR analysis shows miRNA relative expression after knockdown of hsa_circ_0026827.

To illuminate the function of hsa_circ_0026827, we constructed an siRNA expression vector to silence its expression. The results of ALP and ARS staining demonstrated that DPSC osteogenic differentiation decreased 14 days after osteogenic induction in hsa_circ_0026827-silenced cells (Figure 3A). RT-qPCR detection also validated that mRNA expression of osteogenic markers *RUNX1*, *ALP*, *OCN* and *OSX* was significantly decreased during osteogenic differentiation after knockdown of hsa_circ_0026827 (Figure 3B). Dental pulp stem cells were then collected for RNA sequencing (RNA-seq) analyses using hierarchical cluster analysis of miRNA expression. The results revealed that 194 miRNAs were upregulated and 152 were downregulated in knockdown cells (Figure 3C). Heat maps of microarray data showed differential expression of miRNAs, including upregulation of miR-2276-3p, miR-188-3p, miR-2277-3p, miR-210-5p, miR-133a-3p, miR-204-5p, miR-1298-5p, miR-3146, miR-1290 and miR-103a-2-5p (Figure 3D). In particular, RT-qPCR assays indicated that miR-188-3p expression significantly increased after knockdown of hsa_circ_0026827 (Figure 3E).

### Downregulation of miR-188-3p Restored Osteogenic Differentiation of DPSCs After hsa_circ_0026827 Silencing

**FIGURE 4:**
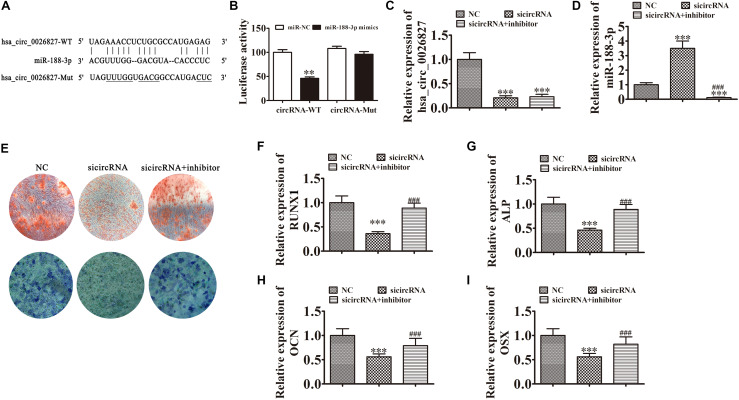
Downregulation of miR-188-3p restored osteogenic differentiation of DPSCs after hsa_circ_0026827 silencing. **(A)** The predicted binding sites of miR-188-3p and hsa_circ_0026827. The mutated (Mut) version of hsa_circ_0026827 is also shown. **(B)** Relative luciferase activity was determined 48 h after transfection with miR-188-3p mimic/normal control (NC) or with wild-type/Mut hsa_circ_0026827 in HEK293T cells. Data are presented as means ± SD. ^∗∗^*P* < 0.01. **(C,D)** RT-qPCR detection shows the expression of hsa_circ_0026827 **(C)** and miR-188-3p **(D)** after transfection with siRNA against hsa_circ_0026827 with and without miR-188-3p inhibitor. Results are presented as means ± SD. ^∗∗∗^*P* < 0.001 vs NC. ^###^*P* < 0.001 vs sicircRNA. **(E)** Photographs of ALP (bottom images) and alizarin red staining (top images) show the osteogenic differentiation potential of DPSCs. **(F–I)** RT-qPCR detection shows the expression of RUNX1, OCN, ALP and OSX. Results are presented as means ± SD. ^∗∗∗^*P* < 0.001 vs NC. ^###^*P* < 0.001 vs sicircRNA.

To illustrate the interactive relationships between hsa_circ_0026827 and miR-188-3p, binding sites between hsa_circ_0026827 and miR-188-3p were predicted using the Starbase web site.^1^ Luciferase vectors containing wild-type or mutated miR-188-3p binding sites were constructed (Figure 4A) and co-transfected into DPSCs with miR-188-3p mimic. Luciferase reporter analysis showed that miR-188-3p inhibited luciferase activity in hsa_circ_0026827 wild-type cells without affecting activity in the mutated cells line, suggesting that miR-188-3p was a potential hsa_circ_0026827 target (Figure 4B). RT-qPCR detection found that hsa_circ_0026827 expression was significantly reduced after hsa_circ_0026827 downregulation, while transfection with miR-188-3p inhibitor did not affect the recovery of hsa_circ_0026827 expression (Figure 4C). RT-qPCR analysis also indicated that silencing hsa_circ_0026827 promoted miR-188-3p expression and treatment with miR-188-3p inhibitor significantly decreased miR-188-3p expression (Figure 4D).

Results of ALP and ARS staining verified that DPSC osteogenic differentiation 14 days after osteogenic induction was decreased after hsa_circ_0026827 silencing (Figure 4E), but downregulation of miR-188-3p restored osteogenic differentiation of DPSCs. RT-qPCR assays also validated that mRNA expression of osteogenic markers *RUNX1*, *ALP*, *OSX*, and *OCN* significantly decreased during osteogenic differentiation after knockdown of hsa_circ_0026827, but miR-188-3p downregulation restored the expression of these markers (Figures 4F–I).

### miR-188-3p Overexpression Suppressed DPSC Osteogenic Differentiation Through Targeting Beclin-1-Mediated Autophagy

**FIGURE 5:**
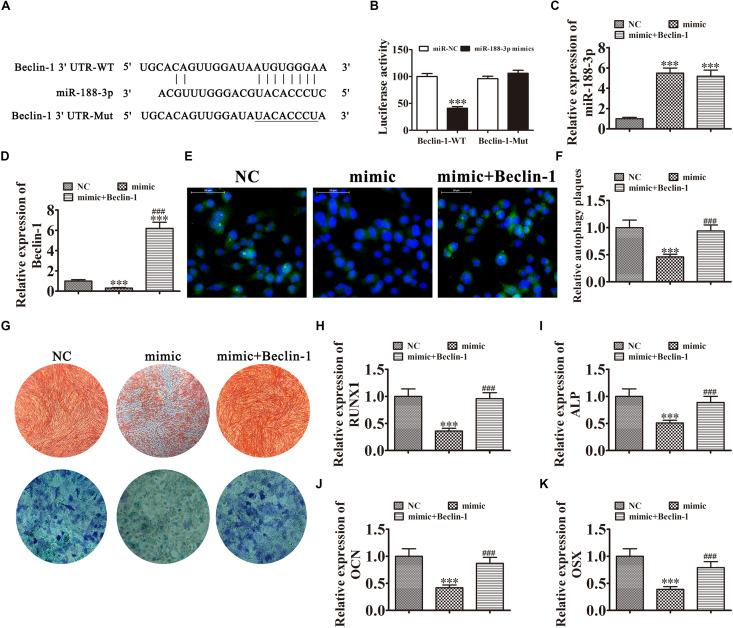
Overexpression of miR-188-3p suppressed osteogenic differentiation of DPSCs by targeting Beclin-1-mediated autophagy. **(A)** The predicted binding sites of miR-188-3p in the 3′UTR of Beclin-1. The mutated (Mut) version of the Beclin-1 3′UTR is also shown. **(B)** Relative luciferase activity was determined 48 h after transfection with miR-188-3p mimic/normal control (NC) or with the wild-type/Mut Beclin-1 3′UTR in HEK293T cells. Data are presented as means ± SD. ^∗∗∗^*P* < 0.001. **(C,D)** RT-qPCR assays show the expression of miR-188-3p (C) and Beclin-1 **(D)** after transfection with miR-188-3p mimic with and without the Beclin-1 overexpression vector. Results are presented as means ± SD. ^∗∗∗^*P* < 0.001 vs NC. ^###^*P* < 0.001 vs mimic. **(E,F)** Immunofluorescence detection shows autophagy plaques. Results are presented as means ± SD. ^∗∗∗^*P* < 0.001 vs NC. ^###^*P* < 0.001 vs mimic. **(G)** Photographs of alizarin red (top images) and ALP staining (bottom images) show the osteogenic differentiation potential of DPSCs. **(H–K)** RT-qPCR analysis shows the expression of RUNX1, OCN, ALP and OSX. Results are presented as means ± SD. ^∗∗∗^*P* < 0.001 vs NC. ^###^*P* < 0.001 vs mimic.

Further experiments found that Beclin-1 was the downstream miR-188-3p target. To examine the relation between Beclin-1 and miR-188-3p, miR-188-3p binding sites in the Beclin-1 3′ untranslated region (3′UTR) were predicted by the Targetscan web site.^2^ We then constructed Beclin-1 luciferase vectors containing wild-type or mutated miR-188-3p binding sites and co-transfected them with miR-188-3p mimic into DPSCs (Figure 5A). miR-188-3p inhibited luciferase activity in cells containing the wild-type Beclin-1 3′UTR but did not affect mutated cell line activity, indicating that Beclin-1 was a potential miR-188-3p target (Figure 5B). RT-qPCR assays found that miR-188-3p expression significantly increased after transfection with miR-188-3p mimic, while transfection with a Beclin-1 overexpression vector did not affect miR-188-3p expression (Figure 5C). RT-qPCR detection also found that miR-188-3p overexpression decreased Beclin-1 expression, but transfection with the Beclin-1 overexpression vector significantly enhanced Beclin-1 expression (Figure 5D). Immunofluorescence assays showed that miR-188-3p overexpression decreased autophagy plaque formation, but after Beclin-1 overexpression, autophagy of DPSCs recovered under osteogenic differentiation conditions (Figures 5E,F).

The results of ALP and ARS staining illustrated that DPSC osteogenic differentiation 14 days after osteogenic induction was decreased by miR-188-3p overexpression (Figure 5G), but Beclin-1 overexpression restored the osteogenic differentiation of DPSCs. RT-qPCR detection also verified that mRNA expression of osteogenic markers *RUNX1*, *ALP*, *OSX*, and *OCN* was significantly decreased during osteogenic differentiation after upregulation of miR-188-3p, but overexpression of Beclin-1 restored expression of these markers (Figures 5H–K).

### miR-188-3p Overexpression Suppressed DPSC Osteogenic Differentiation by Targeting RUNX1

**FIGURE 6:**
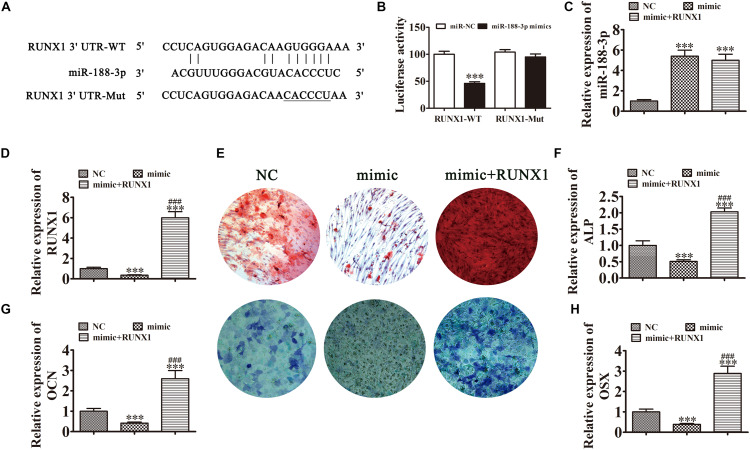
Overexpression of miR-188-3p suppressed osteogenic differentiation of DPSCs by targeting RUNX1. **(A)** The predicted binding sites of miR-188-3p in the 3′UTR of RUNX11. The mutated (Mut) version of the RUNX1 3′UTR is also shown. **(B)** Relative luciferase activity was determined 48 h after transfection with miR-188-3p mimic/normal control (NC) or with the wild-type/Mut RUNX1 3′UTR in HEK293T cells. Data are presented as means ± SD. ^∗∗∗^*P* < 0.001. **(C,D)** RT-qPCR detection shows the expression of miR-188-3p and RUNX1 after transfection with miR-188-3p mimic with **(C)** and without the RUNX1 overexpression vector **(D)**. Results are presented as means ± SD. ^∗∗∗^*P* < 0.001 vs NC. ^###^*P* < 0.001 vs mimic. **(E)** Photographs of alizarin red (top images) and ALP staining (bottom images) show the osteogenic differentiation potential of DPSCs. **(F–H)** RT-qPCR detection shows the expression of OCN, ALP and OSX. Results are presented as means ± SD. ^∗∗∗^P < 0.001 vs NC. ^###^P < 0.001 vs mimic.

In addition to Beclin-1, we determined that RUNX1 was another downstream miR-188-3p target. To examine the interactions between RUNX1 and miR-188-3p, the miR-188-3p binding sites in the RUNX1 3′UTR were predicted through the Targetscan web site tool. We then constructed luciferase vectors containing wild-type or mutated RUNX1 3′UTR sequence (Figure 6A) and co-transfected them with miR-188-3p mimic into DPSCs. Luciferase reporter analysis showed that miR-188-3p inhibited luciferase activity in cells containing wild-type RUNX1 3′UTR without affecting mutated cell line activity, suggesting that RUNX1 was a potential miR-188-3p target (Figure 6B). RT-qPCR analysis found that miR-188-3p expression significantly increased after transfection with miR-188-3p mimic, while transfection with the RUNX1 overexpression vector did not affect miR-188-3p expression (Figure 6C). RT-qPCR assays also indicated that miR-188-3p overexpression decreased RUNX1 expression and that transfection with the RUNX1 overexpression vector significantly increased RUNX1 expression (Figure 6D).

Detection of ARS and ALP showed that the osteogenic differentiation of DPSCs 14 days after osteogenic induction was decreased after miR-188-3p overexpression (Figures 6E,F), but RUNX1 overexpression restored the osteogenic differentiation of DPSCs. RT-qPCR detection also showed that mRNA levels of osteogenic markers *ALP*, *OCN* and *OSX* were significantly decreased during osteogenic differentiation after upregulation of miR-188-3p, but that overexpression of RUNX1 restored the expression of these markers (Figures 6G,H).

### hsa_circ_0026827 Expression Functions Indispensably in Promoting Heterotopic Bone Formation *in vivo*

**FIGURE 7:**
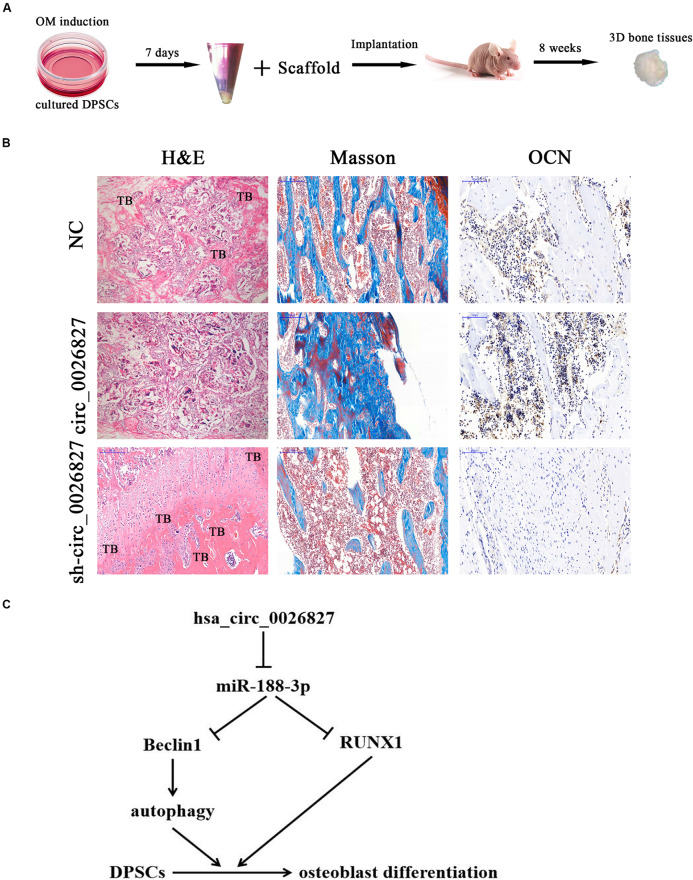
Hsa_circ_0026827 expression plays an important role in promoting heterotopic bone formation *in vivo*. **(A)** Schematic diagram illustrating the experimental setup. **(B)** H&E staining, Masson’s trichrome staining, and immunohistochemical staining of OCN in heterotopic bone. **(C)** Proposed mechanism showing how hsa_circ_0026827 may promote bone formation.

To verify whether hsa_circ_0026827 expression can influence bone formation *in vivo*, we loaded DPSCs expressing sh-hsa_circ_0026827, hsa_circ_0026827 or a negative control (NC) onto Bio-Oss Collagen scaffolds and implanted them in nude mouse subcutaneous tissue (five mice per group). A flowchart of the process is shown in Figure 7A. We harvested implantation samples and analyzed them after 8 weeks. The bone tissue amount shown in H&E staining and collagen organization displaying a blue color by Masson’s trichrome staining was significantly higher in implants containing hsa_circ_0026827 overexpressing cells but were reduced in the sh-hsa_circ_0026827 group. Also, bone trabeculae and osteoblasts were positive for OCN, which can be observed in immunohistochemical staining. The intensity and size of staining increased in the hsa_circ_0026827 overexpression group and decreased in the hsa_circ_0026827 downregulation group (Figure 7B).

## Discussion

An increasing number of studies have found that MSCs exert different but fundamental roles as promoters, enhancers and playmakers of the translational regenerative medicine (for review see Ballini et al., 2017). Recent reports have demonstrated the therapeutic effects of MSCs in animal models, explained by the ability of MSCs to be activated by signals from injured tissues. In these damaged areas, MSCs showed regenerative behavior (Uccelli and Prockop, 2010; Cantore et al., 2018). Studies have also found that cells from dental tissues have an MSC phenotype and can differentiate into osteoblastic cells (Brunetti et al., 2018; Ballini et al., 2019a, b), but the regulatory mechanism is unclear. Previous reports indicated that hsa_circ_0026827 was abnormally expressed during osteogenic differentiation. In the current study, found that hsa_circ_0026827 expression in DPSCs significantly increased during osteogenic differentiation. Downregulation of hsa_circ_0026827 suppressed osteogenic differentiation. In order to clarify the regulatory mechanisms, miRNA expression profiles were analyzed and a number of differentially expressed miRNAs were found, including miR-188-3p. The expression of this miRNA significantly increased after downregulation of hsa_circ_0026827. Luciferase reporter assays validated that miR-188-3p was the target of hsa_circ_0026827. Previous studies have found that miR-188 decreased in osteogenic mouse bone marrow stromal stem cells (Wang et al., 2018). BMSC-specific miR-188 inhibition by intra-bone marrow aptamer-antagomiR-188 injection increased bone formation (Li et al., 2015). This study also verified that evoked miR-188-3p expression impaired cell proliferation, especially tumor cells (Pei et al., 2019; Shi et al., 2019).

To further reveal the regulatory mechanisms, the miR-188-3p target was predicted using a bioinformatics website and luciferase reporter assays. The results verified that miR-188-3p could interact with both the RUNX1 and Beclin1 3′UTRs. Overexpressing miR-188-3p suppressed osteogenic differentiation of DPSCs by targeting RUNX1. The RUNX transcription factor family binds DNA as heterodimers with CBFβ, which function critically in embryonic development. Currently, RUNX3, and RUNX1 have been characterized in the RUNX family (Choi et al., 2001). RUNX1 is a crucial transcription factor that regulates hematopoiesis and hematopoietic stem cells. Increasing evidence has shown that RUNX1 takes part in a variety of maturational processes required for skeletal developmental events (Wang et al., 2005; Qin et al., 2015; Lv et al., 2017). Previous studies found that RUNX1 acts as regulator in BMP9-induced MSCs and that MMC osteogenic differentiation occurs primarily through effects on Smad1/5/8 and MAPK signaling (Rahman et al., 2015).

Our study also found that overexpression miR-188-3p suppressed osteogenic differentiation of DPSCs by targeting Beclin-1-mediated autophagy. Autophagy is a natural self-cannibalization procedure that provides orderly degradation and recycling of dysfunctional cellular organelles or macromolecules to guarantee cellular homeostasis (Sbrana et al., 2016). Studies have illustrated that autophagy functions importantly in stemness and self-renewal of MSC regulation (Rodolfo et al., 2016). Autophagy activation can also stimulate osteogenic differentiation, prevent bone loss and improve the cellular oxidative stress environment at the same time (Gomez-Puerto et al., 2016; Wan et al., 2017; Liang et al., 2019). Our study suggests that hsa_circ_0026827 promotes osteogenic differentiation by upregulating Beclin-1-mediated autophagy through sponging of miR-188-3p.

## Conclusion

This study verified that abnormal hsa_circ_0026827 expression was associated with osteogenic differentiation in DPSCs, which demonstrated that hsa_circ_0026827 promotes osteoblast differentiation of DPSCs via the Beclin1 and RUNX1 signaling pathways by sponging miR-188-3p (Figure 7C). The present study not only furthers our understanding of the role of hsa_circ_0026827 in osteogenic differentiation, but also suggests novel therapeutic possibilities for bone regeneration.

## Data Availability Statement

The raw data supporting the conclusions of this article will be made available by the authors, without undue reservation, to any qualified researcher.

## Author Contributions

FJ, LZ, and JT conceived the research and drafted the manuscript with comments from all authors. JP, ZS, ZY, and JW conducted the experiments and analyses. XB and YL participated in experiments and revised the manuscript. All authors approved the final version.

## Conflict of Interest

The authors declare that the research was conducted in the absence of any commercial or financial relationships that could be construed as a potential conflict of interest.

## Footnotes

1 http://starbase.sysu.edu.cn/

2 http://www.targetscan.org/vert_71/

## Abbreviations

3′UTR: 3′ untranslated region
ALP: alkaline phosphatase
circRNA: circular RNA
DPSCs: dental pulp stem cells
EDTA: ethylene diamine tetraacetic acid
FBS: fetal bovine serum
miRNA: microRNA
MSC: mesenchymal stem cell
OCN: osteocalcin
OSX: osterix
RUNX1: runt-related transcription factor 1.
